# Poor treatment outcomes with palliative gemcitabine and docetaxel chemotherapy in advanced and metastatic synovial sarcoma

**DOI:** 10.1007/s12032-018-1193-5

**Published:** 2018-08-20

**Authors:** Alexandra Pender, Elizabeth J. Davis, Dharmisha Chauhan, Christina Messiou, Omar Al-Muderis, Khin Thway, Cyril Fisher, Shane Zaidi, Aisha Miah, Ian Judson, Winette van der Graaf, Vicki L. Keedy, Charlotte Benson, Robin L. Jones

**Affiliations:** 1 Sarcoma Unit, Department of Medicine, The Royal Marsden NHS Foundation Trust, London, UK; 2 Vanderbilt-Ingram Cancer Center, Nashville, TN USA; 3 Division of Clinical Studies, The Institute of Cancer Research, London, UK

**Keywords:** Synovial sarcoma, Chemotherapy, Gemcitabine, Docetaxel

## Abstract

The outcome for patients with unresectable or metastatic soft tissue sarcoma remains poor with few treatment options. Synovial sarcoma is a rare type of sarcoma, predominantly affecting adolescents and young adults. Following failure of first-line anthracycline-based chemotherapy, several salvage options are available. We reviewed the safety and efficacy of gemcitabine/docetaxel chemotherapy in two tertiary oncology centres. We identified patients treated with gemcitabine/docetaxel between 2004 and 2016 in a UK and a US oncology centre using retrospective pharmacy and medical records. Treatment response, toxicity and outcome data were collected. Twenty one patients were treated with gemcitabine/docetaxel, the majority as a second- or third-line treatment for metastatic disease. The response rate was 5% with a median progression-free survival of 2 months (95% CI 1.3–3.7). Toxicities reported were as expected for this chemotherapy combination. Treatment was not discontinued due to toxicity. Gemcitabine/docetaxel chemotherapy shows little efficacy in synovial sarcoma and should not be offered to this patient group outside a clinical trial context.

## Introduction

Soft tissue sarcomas are a heterogeneous group of rare tumours of mesenchymal origin. They account for about 1% of adult cancers and 15% of paediatric tumours. The mainstay of management for localized disease is complete surgical resection with or without (neo)adjuvant radiation. Despite optimal treatment, approximately 50% of patients with high-grade tumours will develop recurrent or metastatic disease. The outcome of patients with metastatic soft tissue sarcoma is poor with a median overall survival of 12–18 months. First-line chemotherapy for metastatic disease consists of an anthracycline-based regimen [1, 2]. Over the last few years, gemcitabine and docetaxel combination chemotherapy has emerged as an effective salvage schedule, particularly in leiomyosarcoma and undifferentiated pleomorphic sarcoma [3, 4]. In addition, a number of other agents have recently been approved for treating advanced soft tissue sarcomas, including pazopanib, trabectedin, olaratumab and eribulin [5–8].

Synovial sarcoma is an uncommon type of sarcoma, representing approximately 2.5% of all soft tissue sarcomas [9]. This tumour tends to occur in the extremities in adolescents and young adults. It has a characteristic translocation t(X;18;p11.2;q11.2). Retrospective studies have suggested that this subtype is particularly sensitive to ifosfamide chemotherapy [10–12]. However, there are currently few data regarding the role of gemcitabine/docetaxel specifically in synovial sarcoma. Consequently, the aim of this study was to evaluate the efficacy and safety of this combination in synovial sarcoma. We reviewed the use of gemcitabine/docetaxel in relapsed synovial sarcoma in patients from two tertiary cancer centres; at the Royal Marsden Hospital in London and at the Vanderbilt-Ingram Cancer Centre in Nashville, to assess any activity in this setting.

## Methods

Institutional Review Board approval was obtained prior to commencing the study. All patients with a histological diagnosis of synovial sarcoma treated with at least one cycle of gemcitabine/docetaxel chemotherapy between 2004 and 2016 at the Royal Marsden Hospital and 2010 and 2016 at the Vanderbilt-Ingram Cancer Centre were identified using the unit database and pharmacy records. Data regarding baseline characteristics, treatment received, response assessments and treatment toxicities were retrospectively collected from the electronic patient record. Progression-free survival was defined as time from first dose of gemcitabine/docetaxel to date of disease progression and overall survival as time from first dose of gemcitabine/docetaxel to death from any cause. Statistical and Kaplan–Meier analyses were performed using GraphPad Prism version 7.0 (GraphPad Software, La Jolla, California, USA).

An experienced soft tissue pathologist confirmed the diagnosis of synovial sarcoma in all cases. The presence of the t(X;18) translocation was also confirmed. Radiological response was evaluated according to RECIST 1.1 [13]. Response was evaluated after every 2 cycles of therapy. Toxicity was graded according to CTCAE v4.03 [14]. Gemcitabine/docetaxel was administered intravenously on a three-weekly cycle with gemcitabine given at a dose of 540–1000 mg/m^2^ (median 900 mg/m^2^) on Day 1 and 8 of the cycle and docetaxel given at 60–100 mg/m^2^ on Day 8 (median 75 mg/m^2^). The majority of patients were treated according to the gemcitabine/docetaxel schedule detailed in Seddon et al. [15].

## Results

Twenty-one patients were identified across both institutions (Table 1). The median age on commencing gemcitabine/docetaxel was 42 years (range 20–61 years). Eleven of 21 (52%) patients had Grade 3 synovial sarcoma. The majority of patients had previous exposure to doxorubicin/ifosfamide chemotherapy and 19/21 (90%) received gemcitabine/docetaxel as a second- or third-line treatment in the locally advanced or metastatic setting.

**Table 1:** Patient clinical characteristics

Characteristic	*n* (%)
Male	15 (71%)
Female	6 (29%)
Age (years)	
Median	42
Range	20–61
Tumour grade	
1	0
2	2 (10%)
3	11 (52%)
Unknown	8 (38%)
Treatment line in metastatic/advanced setting	
First	1 (5%)
Second	12 (57%)
Third	7 (33%)
Fourth	1 (5%)
Previous exposure to doxorubicin/ifosfamide	
Yes	19 (90%)
No	2 (10%)

Patients received at least one cycle of gemcitabine/docetaxel chemotherapy. The median dose delivered was 900 mg/m^2^ gemcitabine and 75 mg/m^2^ docetaxel, respectively. Treatment was discontinued due to progressive disease or completion of 6 cycles of chemotherapy. Patients received a median of 3 cycles of treatment (range 1–6).

**Fig. 1:**
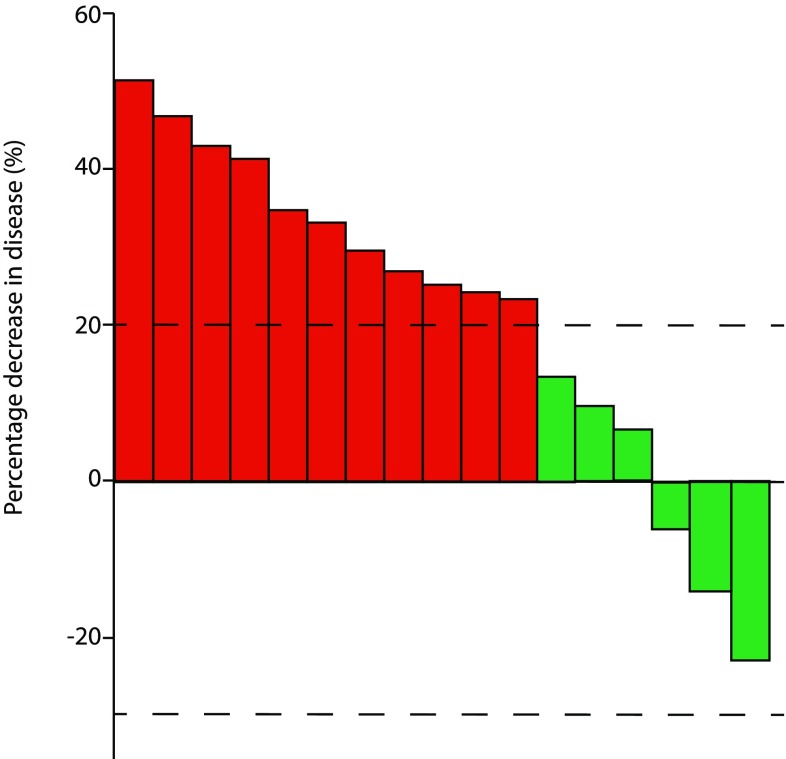
Response to gemcitabine/docetaxel chemotherapy at first response assessment. Red—progressive disease, green—stable disease, dashed lines—limits of stable disease i.e. − 30% and + 20% over baseline measurements

**Fig. 2:**
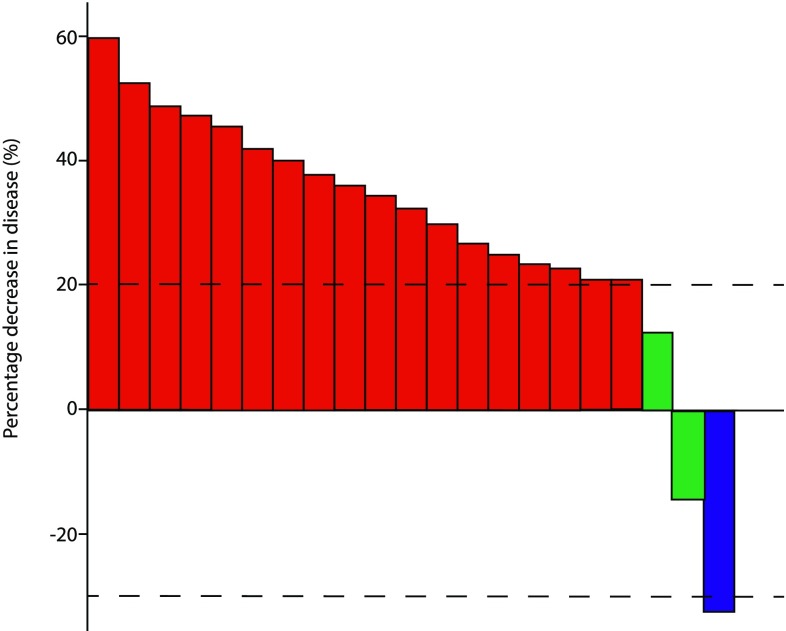
Response to gemcitabine/docetaxel chemotherapy at end of treatment. Red—progressive disease, green—stable disease, blue—partial response, dashed lines—limits of stable disease i.e. − 30% and + 20% over baseline measurements

**Fig. 3:**
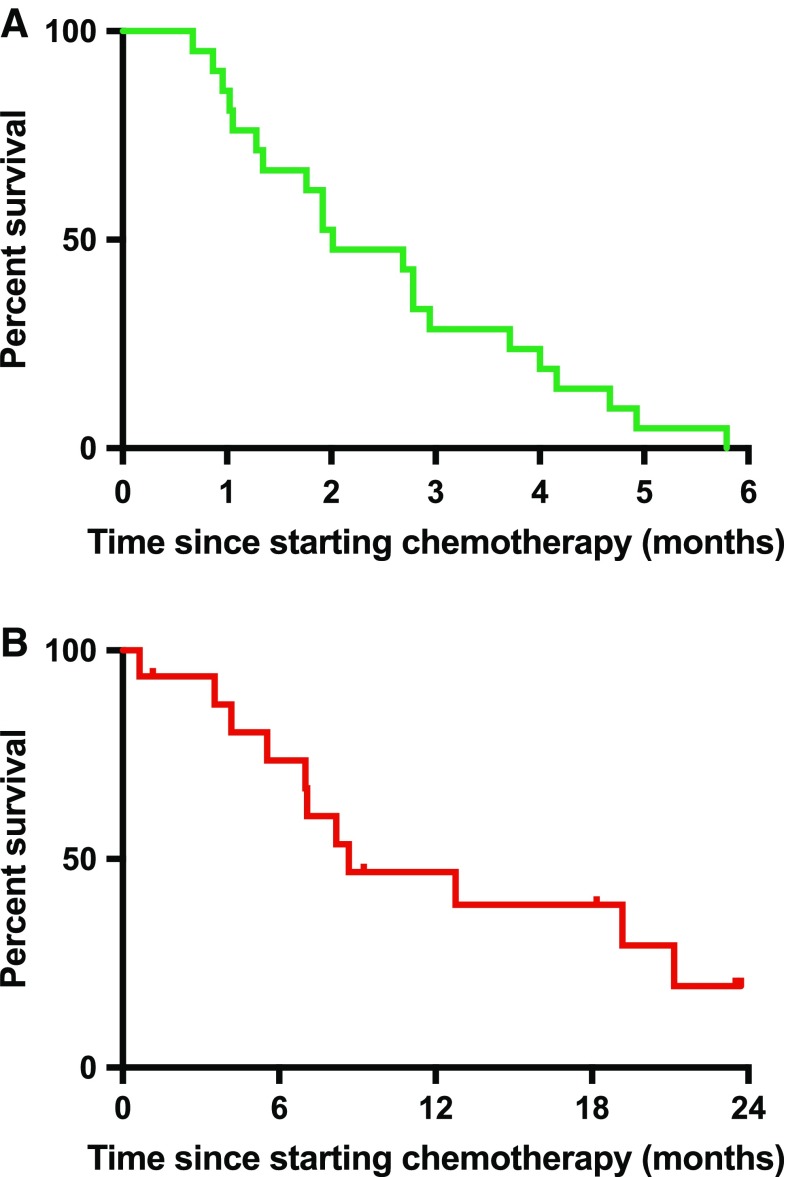
Progression-free survival (**a**) and overall survival (**b**) of synovial sarcoma patient cohort treated with gemcitabine/docetaxel chemotherapy [survival data available for 16/21 (76%) patients]

Response assessments were performed after a median of 2 cycles of treatment for 17 patients and at the end of treatment for all patients. At the interim response assessment, 11/17 (65%) patients had disease progression on imaging (Fig. 1). At the end of the course of treatment, which ranged from 1.5 to 6 cycles of gemcitabine/docetaxel, 18/21 (86%) patients had progressive disease (Fig. 2). One patient had a partial response to treatment after 6 cycles. This patient had biopsy-proven metastatic synovial sarcoma with a known SS18/SSX1 fusion gene on molecular testing of the primary lesion. The median progression-free survival was 2.0 months (95% CI 1.3–3.7, Fig. 3a). Survival data were available for 16/21 patients (76%, Fig. 3b). The median overall survival was 8.4 months (95% CI 6.7–15.1).

Toxicity data were available for 18/21 patients (86%). No patients discontinued treatment due to toxicity. The most frequently reported toxicities were fatigue, anaemia and diarrhoea (Table 2). No Grades 4–5 toxicities were reported.

**Table 2:** Toxicity reported during gemcitabine/docetaxel chemotherapy [available for 18/21 (86%) patients]

Grade	1	2	3
Fatigue	10 (56%)	4 (22%)	1 (6%)
Anaemia	9 (50%)	4 (22%)	1 (6%)
Diarrhoea	3 (17%)	1 (6%)	1 (6%)
Thrombocytopenia	0	1 (6%)	2 (11%)
Leukopenia	2 (11%)	0	0
Neutropenia	1 (6%)	0	0
Nausea	3 (17%)	1 (6%)	0
Vomiting	2 (11%)	0	1 (6%)
Anorexia	1 (6%)	1 (6%)	0
Neuropathy	1 (6%)	1 (6%)	0
Oedema	1 (6%)	1 (6%)	0
Infection	0	2 (11%)	0
ALT increase	0	1 (6%)	0
AST increase	0	1 (6%)	0
Dehydration	0	1 (6%)	0
Rash	0	1 (6%)	0
Abdominal pain	1 (6%)	0	0
Constipation	1 (6%)	0	0
Fever	1 (6%)	0	0
Haemorrhoids	1 (6%)	0	0
Myalgia	1 (6%)	0	0
Palmar-plantar erythrodysesthesia	1 (6%)	0	0

*ALT* alanine aminotransferase, *AST* aspartate aminotransferase

## Conclusion

Gemcitabine and docetaxel combination chemotherapy has emerged as an effective salvage schedule in advanced sarcoma [3, 4, 16], particularly leiomyosarcoma and undifferentiated pleomorphic sarcoma. Synovial sarcoma is generally regarded as a relatively chemosensitive sarcoma subtype, although radiological response assessments in synovial sarcomas are particularly challenging [17]. However, there are few published data regarding gemcitabine/docetaxel in this subtype. Therefore, the aim of this study was to report the utility of this combination in synovial sarcoma. Our results suggest that gemcitabine/docetaxel has little efficacy with a response rate of 5% and median PFS of 2 months.

Recent data have shown no superiority for gemcitabine/docetaxel over doxorubicin chemotherapy in the first-line setting [18]. In a study of 257 treatment-naïve patients with unresectable and metastatic soft tissue sarcoma, the overall response rate to gemcitabine/docetaxel was 20%. 11 patients in this trial had synovial sarcoma; 4% of the patients receiving doxorubicin and 5% of those receiving gemcitabine/docetaxel. A planned analysis by histological subtype suggests that doxorubicin was more active than gemcitabine/docetaxel in these patients [HR 4.15 (1.16–14.85)]. Conversely, the response rate observed in treatment-naïve leiomyosarcoma and relapsed pre-treated metastatic leiomyosarcoma to gemcitabine/docetaxel is approximately 25% [15, 16].

Over the last few years, a number of agents have been approved for the treatment of metastatic soft tissue sarcomas, including olaratumab, pazopanib and trabectedin. The Phase I/II trial of doxorubicin and olaratumab only included 3 synovial sarcoma patients [6]. Consequently, the activity of this agent in synovial sarcoma is unknown. This is likely due to the preference of most oncologists to treat synovial sarcoma patients with combination doxorubicin/ ifosfamide, due to the efficacy of ifosfamide in this subtype [2, 10]. Consequently, it is difficult to comment on the efficacy of olaratumab in synovial sarcoma. In contrast, there are retrospective data to suggest that trabectedin is active in this subtype [12]. The EORTC trials of pazopanib also demonstrate activity in synovial sarcoma [5, 19] as has the Phase II study of regorafenib [20] offering other therapeutic options in this subtype of soft tissue sarcoma.

In conclusion, our study suggests that patients with advanced synovial sarcoma should not routinely be offered gemcitabine/ docetaxel outside the context of a clinical trial. A number of other agents do have activity in this subtype, but further work is required to define the optimal sequence and identify novel therapies.
